# Prognostic Models for Predicting Coronary Heart Disease Risk in Patients with Type 2 Diabetes Mellitus: A Systematic Review and Meta-Analysis

**DOI:** 10.3390/diagnostics16050765

**Published:** 2026-03-04

**Authors:** Maicol Cortez-Sandoval, César J. Eras Lévano, Joaquín Fernández Álvarez, Jorge López-Leal, Lady Morán Valenzuela, Raul H. Sandoval-Ato, Hady Keita, Martin Gomez-Lujan, Fernando M. Quevedo Candela, Jesús I. Parra Prado, José Luis Muñoz-Carrillo, Oriana Rivera-Lozada, Joshuan J. Barboza

**Affiliations:** 1 Escuela de Medicina Humana, Universidad Científica del Sur, Lima 15067, Peru; mcortezs@cientifica.edu.pe; 2 Escuela de Posgrado, Universidad Privada “San Juan Bautista”, Lima 15067, Peru; cesar.eras@upsjb.edu.pe; 3 NeumoVigo I+i Research Group, Galicia Sur Health Research Institute (IIS Galicia Sur), SERGAS-UVIGO, 36312 Vigo, Spain; joaquin.fernandez@uvigo.gal; 4 Instituto Mexicano del Seguro Social, Órgano de Operación Administrativa Desconcentrada Estatal en Chihuahua, Chihuahua 31000, Mexico; jorge.lopezleal22@gmail.com; 5 Escuela de Medicina, Universidad Católica Santiago de Guayaquil, Guayaquil 090615, Ecuador; lady.moran01@cu.ucsg.edu.ec; 6 Escuela de Medicina, Universidad Privada Antenor Orrego, Trujillo 13008, Peru; genrhraul@gmail.com; 7 Universidad de la Sierra Sur, Oaxaca 70800, Mexico; hadykeith@yahoo.fr; 8 Escuela de Medicina, Universidad Nacional Federico Villarreal, Lima 15001, Peru; mgomezl@unfv.edu.pe; 9 Instituto Nacional Cardiovascular, INCOR, Lima 15076, Peru; ferquec206@gmail.com; 10 Escuela de Medicina, Universidad Autónoma de Chihuahua, Chihuahua 31125, Mexico; jimmywessm12@hotmail.com; 11 Laboratorio de Inmunología, Centro Universitario de los Lagos, Universidad de Guadalajara, Lagos de Moreno 47460, Mexico; 12 Vicerrectorado de Investigación, Universidad Señor de Sipán, Chiclayo 14001, Peru; riveraoriana@uss.edu.pe

**Keywords:** coronary heart disease, meta-analysis, prediction model, systematic review, type 2 diabetes mellitus

## Abstract

**Background**: Individuals with type 2 diabetes mellitus (T2DM) are at markedly increased risk of developing coronary heart disease (CHD); however, the generalizability and transportability of existing prediction models remain uncertain. **Objective**: To identify and evaluate multivariable prognostic models developed to predict CHD in adults with T2DM. **Methods**: We conducted a PRISMA-guided systematic review and meta-analysis of multivariable prognostic models predicting CHD in T2DM populations. Model characteristics and performance metrics were extracted following the CHARMS and TRIPOD-SRMA frameworks, and pooled discrimination was estimated on the logit-transformed AUC scale using a random-effects model (REML, Hartung–Knapp adjustment). Between-study heterogeneity and 95% prediction intervals were quantified, while risk of bias and applicability were assessed using the PROBAST tool. **Results**: Thirteen studies encompassing clinical, imaging-based, and omics-augmented models met the inclusion criteria. The pooled AUC was 0.69 (95% CI: 0.66–0.71), with high heterogeneity (I^2^ = 97.4%; τ^2^ = 0.0979) and a wide 95% prediction interval (0.54–0.81). Classical regression-based models demonstrated modest discrimination, whereas machine learning, imaging, and proteomic approaches achieved higher AUC estimates but were frequently constrained by small sample sizes, internal-only validation, and poor calibration reporting. The analysis domain emerged as the principal source of bias in PROBAST evaluations, and applicability issues were most frequent in models requiring advanced imaging or molecular platforms. **Conclusions**: Prognostic models for CHD in T2DM demonstrate moderate-to-good discrimination but substantial heterogeneity and frequent miscalibration across studies. Their clinical utility depends on rigorous external validation and local recalibration, particularly when incorporating imaging or molecular predictors. Future research should prioritize standardized CHD outcomes, consistent calibration reporting, decision-analytic assessments, and the development of transportable multimodal prediction models across diverse populations.

## 1. Introduction

Type 2 diabetes mellitus (T2DM) is closely linked with a markedly elevated risk of developing coronary heart disease (CHD), a condition that continues to be the principal cause of mortality among individuals with T2DM [1,2]. This association highlights the importance of accurately assessing cardiovascular risk in this population, both to guide clinical decision-making and to implement targeted preventive strategies [3]. In response, a variety of prognostic models have been designed to estimate the probability of CHD events specifically in people with T2DM. These models span from widely recognized tools such as the Framingham Risk Score and the UK Prospective Diabetes Study (UKPDS) Risk Engine [4,5] to more advanced approaches that integrate imaging technologies, proteomic data, demographic characteristics, and serum biomarkers. However, the predictive reliability and applicability of these models often vary when tested across different populations. External validations have revealed notable inconsistencies in both discrimination and calibration, particularly when models are applied to cohorts with distinct demographic or clinical profiles. Moreover, a significant proportion of these tools were developed using small sample sizes, lacked external validation, or combined heterogeneous outcomes, limiting their generalizability. Even models like UKPDS-OM2 (Outcomes Model version 2) and RECODe—which are relatively well-established—have produced inconsistent results depending on the outcome assessed and the characteristics of the validation population. Given these limitations, there is a compelling need to consolidate the existing evidence surrounding CHD risk prediction models in people with T2DM. A systematic review and meta-analysis following established guidelines such as CHecklist for critical Appraisal and data extraction for systematic Reviews of prediction Modelling Studies (CHARMS) and PROBAST [6,7] provides an appropriate methodological framework for critically evaluating the development processes, validation approaches, and performance metrics of these models. The present meta-analysis is designed to address this need by systematically identifying and appraising multivariable prognostic models developed for predicting CHD in adults with T2DM. The objective is to evaluate model discrimination and calibration, assess risk of bias, and explore validation strategies, ultimately offering insights to support better risk prediction and clinical application in diverse diabetic populations.

## 2. Materials and Methods

This systematic review and meta-analysis was conducted in accordance with the PRISMA 2020 [8] guidelines and followed the methodological standards outlined in the CHARMS and TRIPOD-SRMA frameworks [9]. The primary objective is to evaluate and synthesise evidence from prognostic model studies aiming to predict the risk of CHD in patients diagnosed with T2DM. The protocol has been registered in the PROSPERO database (CRD420251152663).

### 2.1. Study Design and Eligibility Criteria

Studies were included that developed or validated multivariable prognostic models designed to predict the risk of CHD in adults with T2DM without a history of CHD. The outcome was clearly defined and included myocardial infarction, coronary revascularization (such as angioplasty or bypass surgery), stable or unstable angina, or cardiovascular death. Only original research articles with an observational cohort design, either prospective or retrospective, were considered. Developmental studies were included, which incorporated internal validation techniques such as bootstrap, cross-validation, or data-splitting. External validation studies were included, which assessed model performance in an independent cohort—whether temporal, geographical, or population-based—to determine generalizability. All included studies reported at least one performance metric, such as discrimination (e.g., area under the ROC curve), calibration (slope or intercept), or clinical utility measures, such as net benefit or reclassification indices. Studies focusing on type 1 diabetes mellitus, gestational diabetes, or mixed populations that did not present stratified results for T2DM were excluded. Narrative reviews, editorials, conference abstracts, letters to the editor, and studies conducted in animals or in vitro settings were also excluded. In cases of overlapping study populations, only the most complete and methodologically sound version was retained.

Although inclusion was restricted to coronary heart disease–related outcomes, we anticipated variability in endpoint definitions across studies, ranging from hard coronary events to revascularization procedures and imaging-derived endpoints, which was considered in the interpretation of heterogeneity.

### 2.2. Eligible Population

The target population for this systematic review comprised adults (≥18 years) diagnosed with T2DM, regardless of duration or treatment status, who were at risk of developing CHD. Eligible studies included participants with a confirmed diagnosis of T2DM according to established clinical or biochemical criteria (e.g., ADA or WHO definitions) and did not restrict inclusion to specific subgroups, such as individuals with established CHD at baseline, gestational diabetes, or other forms of secondary diabetes. Studies that included mixed diabetic populations were only considered if they presented stratified outcomes specific to T2DM.

The outcomes of interest were related to the development of CHD, defined by clinical endpoints such as myocardial infarction, angina pectoris, coronary revascularization (percutaneous or surgical), or cardiovascular mortality. Studies in which CHD was operationalized solely through diagnostic imaging without clinical correlation were included only if their prognostic relevance was explicitly addressed. No restrictions were placed on geographic location, sex distribution, or ethnicity of the study population, although these factors were considered in the heterogeneity analysis.

### 2.3. Candidate Prognostic Factors

The prognostic models identified in this review were expected to incorporate a broad range of candidate predictors that have been empirically or mechanistically linked to the risk of CHD in individuals with T2DM. These predictors could be clinical, biochemical, genetic, or imaging-based. Among the most consistently evaluated factors were classical cardiovascular risk variables such as age, sex, smoking status, duration of diabetes, systolic and diastolic blood pressure, and body mass index (BMI).

Metabolic and inflammatory biomarkers, including glycated hemoglobin (HbA1c), total cholesterol, low-density lipoprotein cholesterol (LDL-C), high-density lipoprotein cholesterol (HDL-C), triglycerides, and markers of systemic inflammation such as C-reactive protein (CRP) and neutrophil-to-lymphocyte ratio (NLR), were carefully examined. Emerging proteomic biomarkers such as PCSK9, CD27, and NRP1, as well as large endothelin-1 (Big ET-1), were also evaluated for inclusion in validated models and their relative contribution to predictive accuracy. Studies incorporating composite indices such as non-HDL cholesterol, the wide red blood cell-to-albumin distribution width (RAR), or multi-omics data were also included when reported in a prognostic modeling context.

Each predictor was extracted based in terms of its definition, measurement method, unit of analysis, timing of assessment, and whether it was retained in the final model. The frequency and consistency of inclusion across models were documented, and where possible, the relative prognostic weight or effect size was reported. This review provides a comparative synthesis of prognostic factors across different studies to determine which predictors consistently demonstrate strong and independent associations with the risk of CHD in populations with T2DM.

### 2.4. Search Strategy

A comprehensive electronic search was conducted in MEDLINE (via PubMed), Embase, Scopus, and Web of Science to identify relevant studies from inception to April 2025. The search strategy employed a combination of controlled vocabulary and free-text terms related to (“type 2 diabetes mellitus”), (“coronary heart disease”), (“prediction model”), (“risk score”), (“prognostic”), and (“validation”). No language restrictions were applied. In addition, the reference lists of all included relevant articles and reviews were reviewed to identify further eligible studies. The complete search strategy for each database is included in the Supplementary Material.

### 2.5. Study Selection Process

All retrieved records were uploaded into EndNote 20 for de-duplication and then imported into the Rayyan platform for systematic screening. The selection process was conducted in two phases: an initial screening of titles and abstracts, followed by a full-text review. Two reviewers trained in systematic review methodology performed both stages independently. Discrepancies were resolved through discussion and, when necessary, by a third reviewer. A PRISMA flow diagram was used to document the screening process and justify exclusions at each stage.

### 2.6. Data Extraction and Management

Data extraction was performed independently by two reviewers using a standardized and pilot-tested form developed based on the CHARMS checklist. Extracted data included general study characteristics (authors, year, country, setting), participant characteristics (age, sex, diabetes duration, comorbidities), model specifications (type of model, predictors used, method of selection, modeling techniques), outcome definitions and follow-up duration, performance metrics (e.g., AUC, calibration slope, Brier score), and the nature of internal or external validation (e.g., split-sample, bootstrapping, temporal or geographical validation). Where necessary, authors of primary studies were contacted to obtain missing or incomplete information.

### 2.7. Risk of Bias and Applicability Assessment

To evaluate the methodological quality and applicability of each included study, the Prediction Model Bias Risk Assessment Tool (PROBAST) was used [10]. This tool assesses risk of bias across four domains: participants, predictors, outcome, and analysis. Each domain was rated as low, high, or unclear risk of bias, and an overall judgment was provided for each model. Applicability was also assessed based on the relevance of the study population, predictors, and outcomes to the review question. Two reviewers conducted the assessment independently, and discrepancies were resolved by consensus.

To improve clarity and facilitate interpretation of the PROBAST assessments across multiple domains, a standardized color-coding scheme was applied. Each background color corresponds directly to the categorical judgments defined by the PROBAST tool and was used consistently across all risk-of-bias and applicability domains.

Specifically, green indicates low risk of bias or low concern for applicability, yellow indicates some concerns, and redindicates high risk of bias or high concern for applicability. This visual representation was implemented solely to enhance readability and allow rapid identification of methodological patterns across studies.

### 2.8. Data Synthesis and Statistical Analysis

A random-effects meta-analysis was conducted to synthesize the discriminative performance of prognostic models for predicting CHD in individuals with T2DM. The primary outcome of interest was the area under the receiver operating characteristic curve (AUC), extracted or derived from studies reporting external validation of prognostic models in at least two independent cohorts. To account for the asymmetry and bounded nature of the AUC scale, reported AUC values were logit-transformed prior to pooling. The meta-analysis was performed using the Metagen function from the {meta} package in R (version 4.3.0), employing a random-effects model with restricted maximum likelihood (REML) estimation and Hartung–Knapp adjustments. Between-study heterogeneity was assessed using the I^2^ statistic and τ^2^ estimates. A 95% prediction interval was computed to evaluate the potential variability in performance across future settings.

Given the expected conceptual heterogeneity across prognostic model classes (clinical scores, imaging-augmented, omics/genetic, and machine-learning approaches), we pre-specified the quantitative synthesis of AUC as an exploratory summary of discriminative performance rather than a clinically transportable estimate. Accordingly, narrative synthesis was prioritized to contextualize performance according to model purpose, predictor requirements, and intended care pathway, and subgroup analyses were used to partially account for differences in model class.

The pooled logit (AUC) was then back transformed to the original AUC scale to facilitate clinical interpretation. Study-specific logit (AUC) values and standard errors were also back-transformed, and a forest plot was constructed using ggplot2 to display the AUC values and corresponding 95% confidence intervals visually. The reference line at AUC = 0.5 was added to denote chance-level discrimination. The pooled AUC across studies was 0.69 (95% CI: 0.66 to 0.71), indicating the included prognostic models’ good discriminative ability. The substantial between-study heterogeneity (I^2^ = 97.4%, τ^2^ = 0.0979, *p* < 0.0001) suggests that model performance varies across settings and populations. A prediction interval of [0.54 to 0.81] was also estimated on the logit scale, highlighting the potential spread of future AUC values.

Calibration performance was not pooled quantitatively due to inconsistent reporting and definitional variability; instead, it was summarized narratively. Future efforts should focus on standardizing calibration reporting and improving transparency in model validation.

## 3. Results

### 3.1. Selection of Studies

**Figure 1:**
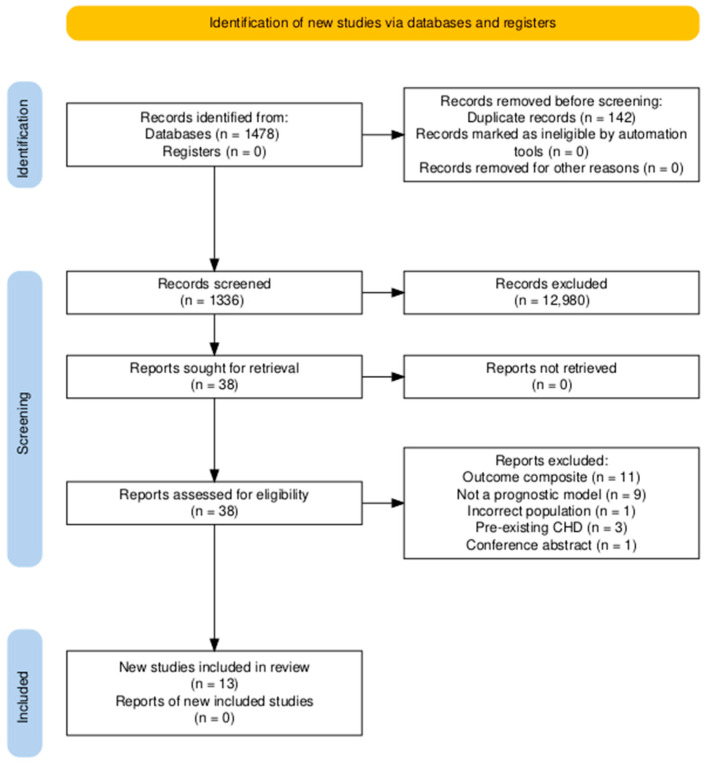
PRISMA flow chart of selection studies.

Our search identified 1478 records through database searches (PubMed, n = 64; Scopus, n = 1237; Web of Science, n = 123; Embase, n = 54). After removing 142 duplicates, 1336 records were screened by title/abstract, and 1298 were excluded. Thirty-eight full-text reports were assessed for eligibility; 25 were excluded for the following reasons: outcome composite (n = 11), not a prognostic model (n = 9), incorrect population (n = 1), pre-existing CHD (n = 3), and conference abstract (n = 1). Thirteen studies met the inclusion criteria and were included in the review (Figure 1) [11,12,13,14,15,16,17,18,19,20,21,22,23].

### 3.2. Characteristics of Included Studies

A total of 13 prognostic models externally validated in T2DM populations were identified and evaluated (Table 1). The studies spanned multiple countries, including the United Kingdom, the United States, France, Germany, China, Japan, South Korea, and Malaysia, with publication years ranging from 2005 to 2025. The target population studies comprised adults with T2DM, often without a prior history of cardiovascular disease (CVD), although some studies specifically included high-risk individuals. Sample sizes varied substantially, from fewer than 200 participants to over 20,000.

The predicted outcome in the studies was primarily CHD, encompassing both acute and chronic presentations such as myocardial infarction and stable or unstable angina. However, our focus was restricted to studies that reported CHD as an outcome. We excluded studies that evaluated only composite endpoints—such as major adverse cardiovascular events (MACE)—in which CHD was not reported as an individual outcome (e.g., those including only heart failure, stroke, or other non-coronary events). Some models also examined additional outcomes, including all-cause mortality, heart failure, nephropathy, or stroke, but these were not considered in the primary analyses.

Outcome definitions varied substantially across studies, including hard CHD events (myocardial infarction and coronary death), revascularization-based endpoints, composite cardiovascular outcomes, and, in imaging-focused studies, surrogate or intermediate measures, contributing to between-study heterogeneity.

The predictors used in the models included traditional clinical and laboratory variables (e.g., age, sex, blood pressure, HbA1c, lipids) [24], imaging-derived measures (e.g., coronary artery calcium score [CACS], myocardial perfusion entropy) [25] and, in more recent models, proteomic biomarkers [26]. Modeling approaches were diverse, ranging from traditional regression techniques (e.g., Cox regression, logistic regression) to more complex machine learning methods (e.g., random forests, gradient boosting, LASSO, neural networks). Several studies compared multiple modeling techniques within the same cohort. Validation methods varied, with some models undergoing internal and external validation using independent datasets, while others only performed split-sample or cross-validation. Performance was most frequently reported using the C-statistic or AUC, with values ranging from 0.50 to 0.83. In most studies, inclusion of advanced predictors or machine learning approaches led to modest improvements in discrimination. Calibration metrics were inconsistently reported but indicated variability across models. Notably, the RECODe model [27] and UKPDS Outcomes Model 2 (OM2) [28] were frequently evaluated and served as comparators in several studies. Overall, the heterogeneity in model predictors, populations, and validation methods underscores the variability in performance and the need for context-specific application. Several models demonstrated good discrimination, but overprediction and poor calibration were frequent, especially when applied to contemporary T2DM populations differing from the original derivation cohorts.

### 3.3. PROBAST Assessment in Included Studies

The PROBAST assessment represents co-primary evidence alongside quantitative performance metrics, providing critical insight into the credibility and transportability of reported model performance.

Across development studies, the analysis domain emerged as the predominant source of high risk of bias, particularly among machine-learning and omics-based models, driven by internal-only validation, limited events per predictor, and incomplete calibration reporting.

Using PROBAST, most studies were methodologically acceptable with respect to who was included and what was measured, but the analysis domain was a consistent weak point (Table 2). Populations were generally well described and appropriate for the review question, particularly in large, population-based cohorts and pragmatic trial datasets. Concerns arose mainly in highly selected settings—such as surgical candidates, hospital high-risk clinics, or asymptomatic individuals undergoing coronary CT angiography—where spectrum effects and referral pathways limit representativeness for routine T2DM care. Predictors were usually routine clinical and laboratory variables (age, sex, blood pressure, HbA1c, lipids), yielding low concern for measurement and availability. Issues surfaced when models depended on non-routine predictors—advanced imaging, large proteomic panels, or ancestry-dependent polygenic scores—where platform dependence, standardization, and resource needs complicate real-world implementation and raise applicability concerns. Outcomes were typically “hard” coronary events (MI, coronary death, revascularization) with clear definitions and ascertainment; uncertainty was more likely when composite endpoints mixed coronary with non-coronary events or when adjudication procedures were insufficiently detailed.

Across studies, the analysis domain drove most of the risk of bias. Development papers—especially those using machine learning, imaging, or proteomics—frequently relied on internal validation only (split-sample or k-fold cross-validation), with limited events per predictor, data-driven variable selection without adequate penalization, incomplete handling of missing data, and sparse reporting of calibration. These features are prone to optimism in apparent performance and undermine transportability. By contrast, large external validations of legacy scores were stronger analytically and more transparent but often revealed miscalibration (typically overprediction) when models were applied to contemporary T2DM cohorts, underscoring the need for recalibration or model updating.

Risk of bias assessment using PROBAST was considered co-primary evidence alongside quantitative performance metrics. In particular, a high risk of bias in the analysis domain was consistently identified, especially among machine-learning, imaging-based, and omics-driven models. This was primarily driven by reliance on internal-only validation strategies, limited events per predictor, data-driven variable selection, and incomplete calibration assessment. Consequently, apparent gains in discriminative performance were interpreted cautiously, as they are likely influenced by optimism bias, restricted external validation, and feasibility constraints that limit transportability to routine clinical settings.

### 3.4. Meta-Analysis of Prognostic Models

In the pooled analysis of prognostic models for CHD in patients with T2DM, the overall discriminative performance was moderate to good.

**Figure 2:**
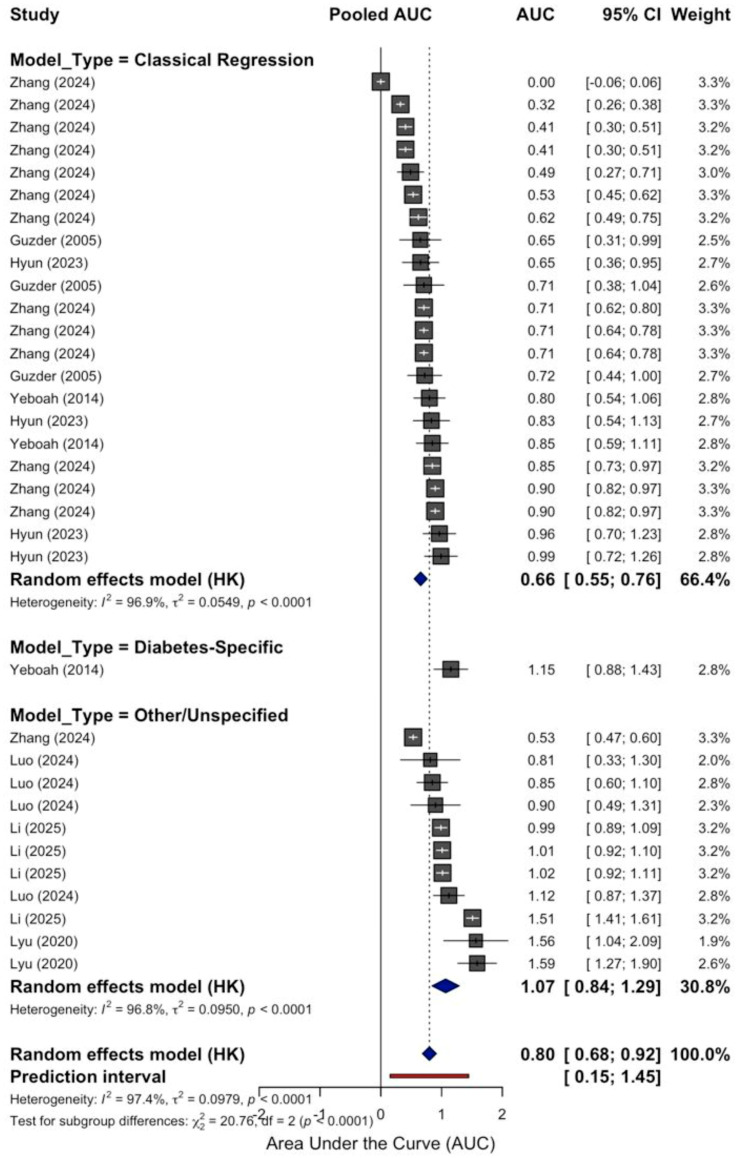
Forest Plot of AUC and 95%CI. Pooled analysis of prognostic models for CHD in patients with T2DM [11,12,13,14,17,21,22,23].

The random-effects model yielded a pooled AUC of 0.69 (95% CI 0.66–0.71), with substantial heterogeneity (I^2^ = 97.4%) and a wide prediction interval (0.54–0.81), indicating marked variability in performance across settings. Accordingly, the pooled estimate should be interpreted cautiously as an exploratory summary rather than a directly generalizable performance measure (Figure 2).

Given the substantial clinical, methodological, and conceptual heterogeneity across included studies, the pooled AUC should be interpreted strictly as an exploratory summary of discriminative performance rather than as a clinically transportable estimate. In this context, discrimination alone is insufficient to support clinical implementation. Consequently, pooled AUC values should not be interpreted as evidence for direct clinical adoption of any prognostic model without prior local external validation, appropriate recalibration to baseline risk and treatment patterns, and demonstration of clinical utility through decision-analytic or impact studies.

Subgroup analyses by model type revealed notable differences. Models classified as Classical Regression (e.g., Framingham and UKPDS-derived scores) contributed the largest proportion of studies and showed a pooled AUC of 0.66 (95% CI 0.63–0.68), suggesting only modest discrimination in this population. In contrast, Diabetes-Specific models were underrepresented, with only one eligible study [12], which reported an AUC of 0.76 (95% CI 0.71–0.81); however, the limited evidence precludes robust conclusions. Models grouped under Other/Unspecified, which encompassed mixed or machine learning approaches, showed higher pooled discrimination (AUC 0.74, 95% CI 0.70–0.78), although heterogeneity remained considerable (I^2^ = 96.8%). The test for subgroup differences was statistically significant (χ^2^ = 20.76, df = 2, *p* < 0.0001), indicating that part of the variability may be explained by the type of model employed. Variability in endpoint definitions likely contributed to the observed heterogeneity and limits the clinical interpretability of pooled discrimination estimates across studies.

Because included studies addressed different clinical questions and care pathways and used heterogeneous predictor sets, the pooled AUC should be interpreted as an exploratory quantitative snapshot rather than a summary applicable to any single implementation context.

Taken together, these findings underscore that while prognostic models for CHD in T2DM demonstrate potentially useful discriminative capacity, their performance varies substantially across methodological approaches and populations. Classical regression-based scores appear to underperform compared to newer approaches, but the latter are often limited by small sample sizes, potential overfitting, and insufficient external validation. The wide prediction interval emphasizes the importance of local validation before clinical implementation.

## 4. Discussion

### 4.1. Principal Findings

Across 13 external validations and model developments in people with T2DM, discrimination for CHD-related outcomes was generally modest-to-good. The pooled analysis yielded an overall AUC of 0.69, but with extreme between-study heterogeneity.Given the extreme between-study heterogeneity, the pooled AUC should be interpreted as an exploratory summary of discriminative performance rather than a clinically transportable estimate, with the wide prediction interval underscoring substantial context dependency [11,12,15,16,21,23]. Machine-learning models rarely outperformed strong regression baselines by large margins, and gains were more consistent when models incorporated richer phenotyping (coronary imaging, liver phenotype) or high-dimensional omics [14,17,18,19,20,22]. Transportability remained a critical fault line: model performance and calibration varied substantially across cohorts and care settings, even for widely used tools [13,23].

A key methodological consideration is that the included models are conceptually heterogeneous, spanning classical clinical scores, imaging-augmented tools, and omics/ML approaches that are designed for distinct clinical pathways. We therefore interpret the pooled AUC primarily as an exploratory descriptor of the overall discriminative landscape and as a vehicle to quantify variability (e.g., prediction intervals), rather than as evidence of generalizable performance. The primary contribution of this review lies in the context-specific narrative synthesis and the PROBAST-based appraisal of model credibility and implementation constraints across model classes.

Despite efforts to focus on coronary heart disease outcomes, substantial variability in endpoint definitions across studies remains an important limitation. Some models targeted hard coronary events, whereas others incorporated revascularization, composite cardiovascular endpoints, or surrogate imaging-based measures. These differences reflect distinct clinical questions and care pathways and likely contribute to the extreme heterogeneity observed. Consequently, pooled discrimination metrics should not be interpreted as reflecting performance for a single, uniform CHD outcome.

Importantly, increased algorithmic or molecular complexity should not be interpreted as evidence of clinical superiority per se. In the absence of robust external validation, adequate calibration, and demonstrated impact on clinical decision-making, apparent improvements in discriminative performance remain insufficient to justify clinical implementation. Furthermore, heterogeneity in outcome definitions and endpoint operationalization across studies reflects differences in clinical questions and care pathways and represents a major contributor to the substantial between-study heterogeneity observed in quantitative synthesis.

### 4.2. Classic Diabetes-Specific Versus General Cardiovascular Scores

Early work comparing UKPDS [29] and Framingham [30] in newly diagnosed T2DM already signaled imperfect transportability: both tools stratified risk but showed limitations when applied outside their derivation era and setting [11]. More recently, UKPDS-OM2 performed sub-optimally in a contemporary UK trial cohort, highlighting calibration drift and emphasizing the need to re-estimate or recalibrate legacy models for present-day care and therapeutics [13]. By contrast, large-scale external validation in UK Biobank suggested that population-based scores such as QRISK/Score can show respectable discrimination in T2DM, but miscalibration is common and clinically meaningful [23]. Together, these data argue that neither “diabetes-specific” nor “general CVD” equations are plug-and-play across T2DM populations without local updating.

### 4.3. What Do Machine-Learning Models Add?

Although several machine-learning and omics-based models report higher point estimates of discrimination, these incremental gains are generally modest, inconsistent across outcomes, and rarely supported by independent external validation. Importantly, algorithmic complexity should not be conflated with clinical superiority, as many of these models rely on internal-only validation and lack robust calibration assessment, increasing susceptibility to optimism bias.

Moreover, the reliance on resource-intensive predictors, specialized platforms, and limited availability of omics or advanced imaging data substantially constrains the feasibility of these models in routine clinical practice.

Aminian et al. developed individualized 10-year risk tools for surgical and non-surgical T2DM populations; their best AUCs were ~0.79–0.81 for mortality and ~0.66–0.67 for coronary events, with calibration curves close to ideal. In head-to-head testing, the IDC models outperformed RECODe among non-surgical patients, but ML only modestly surpassed regression in select endpoints [16]. A national study from Malaysia applied ML to predict diabetes complications, reporting respectable discrimination using routine clinical data, again underscoring that careful feature curation and validation often matter more than the specific algorithm [15]. Overall, ML helped operationalize multi-outcome calculators and improved usability but did not consistently deliver large accuracy jumps over well-specified regression.

Importantly, apparent gains in discrimination observed in machine-learning and molecular models should be interpreted in light of PROBAST findings, as high risk of bias in the analysis domain suggests that optimism bias likely contributes to their reported superiority.

Consistent with PROBAST findings, the apparent superiority of some machine-learning and molecular models is likely influenced by high risk of bias in the analysis domain rather than by true improvements in generalizable prognostic performance.

### 4.4. Imaging and Organ-Specific Phenotyping

Several studies demonstrate that imaging enriches risk stratification in T2DM beyond traditional factors. A cohort of asymptomatic T2DM followed for ~11 years showed that coronary CT angiography (CCTA) provided strong long-term prognostic information for cardiac death and myocardial infarction [21]. SPECT myocardial perfusion “entropy”—a texture/heterogeneity metric—carried independent prognostic value in high-risk T2DM, highlighting microvascular/perfusion heterogeneity as a pathophysiologic signal [19]. Non-alcoholic fatty liver disease (NAFLD), assessed in patients with suspected CAD, was also independently associated with future cardiovascular events, suggesting hepatic phenotype as an accessible enrichment marker [18]. Earlier development work combining MESA and HNR cohorts showed that adding coronary artery calcium (CAC) to a diabetes-specific CHD tool materially improved discrimination and reclassification, especially in men (2AUC~0.73–0.79) [12]. Collectively, these results suggest that an “organ-informed” approach (coronary and hepatic phenotypes) can meaningfully sharpen CHD risk estimates in T2DM.

### 4.5. Molecular Risk: Proteomics and Polygenic Scores

Two large proteomic studies found that circulating protein signatures in T2DM are strongly associated with incident CHD, with evidence that proteomic signals mediate part of the diabetes–CHD link and may support individualized risk assessment [14,22]. A complementary line of evidence shows that a CHD polygenic risk score (PRS) meaningfully improves discrimination and reclassification in T2DM even after adjustment for traditional risk factors and therapies (statins, antihypertensives, glucose-lowering drugs) [13]. Integration pathways remain a gap: we still lack pragmatic frameworks that blend PRS and proteomic signals with clinical and imaging features in transportable models, and few studies test clinical utility via decision-curve or impact analyses.

### 4.6. Context Matters: Who Is the Model for?

Risk equations performed differently across: (i) newly diagnosed vs. longstanding T2DM; (ii) asymptomatic screening vs. suspected CAD; (iii) surgical vs. usual care cohorts; and (iv) Asian vs. Western settings [11,15,16,19,20]. For instance, the IDC models were built explicitly to contrast surgical and non-surgical trajectories, while CCTA studies targeted asymptomatic individuals [16,19]. A model for “average clinic patients” without imaging will not be the right tool for a surgical decision or for asymptomatic screening; conversely, image-augmented models may be poorly transportable to primary care without access to CCTA or CAC. These observations reinforce the need for model-to-task alignment and for impact studies that demonstrate net benefit in the intended workflow.

### 4.7. Calibration, Updating, and Transportability

From a clinical perspective, usefulness depends on accurate absolute risk estimation, appropriate calibration, and demonstrated impact on decision-making, rather than discrimination alone. Across included studies, calibration metrics such as calibration slope, intercept, or calibration-in-the-large were inconsistently reported, precluding quantitative synthesis and limiting clinical interpretability. Consequently, even models with acceptable or high AUC values cannot be assumed to be clinically useful without adequate calibration within the intended care pathway.

Although several studies reported decision-analytic metrics such as NRI, IDI, or net benefit, these measures were not quantitatively synthesized due to heterogeneity in definitions, thresholds, and reference models, which precluded meaningful pooling.

Across validations, miscalibration was frequent and clinically relevant [13,23]. Even when discrimination was acceptable, risk estimates often required recalibration to local outcome rates and treatment patterns. The UKPDS-OM2 [20] example in a modern trial cohort is instructive: therapy evolution (e.g., statins, SGLT2i/GLP-1RA uptake), risk factor control, and case-mix drift can erode the transportability of legacy tools; periodic updating and local recalibration should be the rule, not the exception [13].

Importantly, PROBAST assessments should be considered co-primary evidence alongside quantitative synthesis, as high risk of bias in the analysis domain—particularly among ML-, imaging-, and omics-based models—likely contributes to optimism in apparent performance estimates.

Accordingly, quantitative discrimination metrics and PROBAST-based risk-of-bias assessment should be interpreted jointly, as apparent performance advantages are not clinically meaningful when methodological credibility is compromised.

### 4.8. Where Are the Biggest Gaps?

First, heterogeneity of outcomes and predictors still complicates synthesis: many models target composite MACE, while relatively fewer report CHD-specific discrimination with full uncertainty (CIs) and calibration plots [16,23]. Second, few studies evaluate net clinical benefit or decision impact; most stop at AUC and internal/external validation [16]. Third, high-dimension markers (imaging, omics, PRS) show promise, but we lack head-to-head comparisons that test incremental value on top of robust clinical baselines, and we have limited evidence in underrepresented regions and ethnic groups [12,14,15,17,18,20,22]. Finally, consistent handling of missing data, transparent feature selection, and prospective impact studies remain under-delivered relative to current reporting standards.

Beyond discrimination, calibration emerged as a critical and consistently underreported limitation across studies. Although several models demonstrated acceptable AUC values, incomplete reporting of calibration metrics—such as calibration slope, intercept, or calibration-in-the-large—precluded quantitative synthesis and limits clinical interpretability. As absolute risk estimation is fundamental for clinical decision-making, the absence of standardized calibration reporting represents a major barrier to real-world implementation, regardless of discriminative performance.

From a broader methodological perspective, these findings align with prior evidence-synthesis work conducted by our group, which consistently demonstrates that high between-study heterogeneity, incomplete reporting of calibration, and reliance on single performance metrics substantially limit the clinical interpretability and transportability of predictive models across diverse settings. Across different clinical domains, our previous systematic reviews and meta-analyses have highlighted the importance of integrating quantitative synthesis with structured risk-of-bias assessment, transparent reporting standards, and context-aware interpretation to avoid overgeneralization of pooled estimates. Collectively, this body of work reinforces that robust methodological appraisal and cautious interpretation are essential prerequisites for translating prognostic models into meaningful clinical decision support [31,32,33,34].

## 5. Conclusions

Increased algorithmic or molecular complexity should not be interpreted as evidence of clinical superiority in the absence of robust external validation, adequate calibration, and demonstrated impact on clinical decision-making.

In this systematic review of prognostic models for CHD in people with T2DM, average discrimination was moderate-to-good, but performance varied widely across studies and settings. Classical regression tools—often the most extensively validated—showed modest discrimination and frequent miscalibration in contemporary cohorts, underscoring the need for local updating. Newer approaches that incorporate machine learning, imaging, proteomics, or polygenic scores can raise point estimates of discrimination, yet these gains are tempered by high between-study heterogeneity, analysis-domain bias (internal-only validation, limited events per predictor, optimism), and practical constraints that limit transportability.

Therefore, pooled discrimination metrics should not be interpreted as supporting direct clinical adoption of any model class without prior local validation, recalibration, and demonstration of decision-analytic benefit.

From a methodological standpoint, PROBAST highlighted the analysis domain as the principal weak point. Many development studies did not provide independent external validation, robust handling of missing data, shrinkage/penalization, or thorough calibration assessment. By contrast, large external validations in population-based samples were more robust but consistently revealed calibration drift of legacy scores—emphasizing that periodic recalibration should be standard practice.

For clinical use, a stepwise strategy is warranted: begin with a strong, locally recalibrated clinical model; add higher-yield enhancers (e.g., CAC/CCTA, hepatic phenotype) in appropriate contexts; and consider molecular predictors (proteomics, PRS) only where platforms, ancestry considerations, and workflow integration permit. Implementation should be contingent on local validation and demonstrated decision-analytic benefit.

Future research should prioritize (i) standardized CHD endpoints with full reporting of discrimination and calibration (including slope and calibration-in-the-large), (ii) pre-registered, independent external validations with explicit updating strategies, (iii) decision-curve and impact analyses to prove clinical usefulness, and (iv) transparent, updateable multimodal models evaluated across diverse regions and ancestries. Until such evidence is routine, model adoption in T2DM should proceed with careful local validation, recalibration, and clear acknowledgment of setting-specific limitations.

## Figures and Tables

**Table 1:** Prognostic models externally validated in T2DM populations (CHARMS checklist).

Author	Year	Country	Study Design	Population Participant Eligibility and Recruitment Method (Inclusion Criteria)	Outcome Definition and Method for Measurement of Outcome	Predictors Number and Type of Predictors (e.g., Demographics, History, Lab Results)	Model Type Modelling Method (e.g., Logistic, Survival, Neural Network, Machine Learning)	Validation In Case of Poor Validation, Was the Model Adjusted or Updated?	Performance Calibration and Discrimination Measures with Confidence Intervals
Mohamad Zulfikrie Abas et al. [15]	2025	Malasia	Retrospective cohort	T2D patients from 172 public health clinics in Southern Malaysia	All-cause mortality, retinopathy, nephropathy, ischemic heart disease (IHD), cerebrovascular	Demographics, clinical parameters (e.g., BP, HbA1c, LDL), medical history	Seven algorithms tested (LR, SVM, kNN, DT, RF, XGB, LGBM); LGBM performed best	Evaluated thresholds for optimal balance; model interpretability assessed through feature importance	LGBM ROC-AUC: Mortality 0.84, Retinopathy 0.71, Nephropathy 0.71, IHD 0.66, CeVD 0.74
Ali Aminian et al. [16]	2020	United States	Retrospective cohort	2287 T2D patients undergoing metabolic surgery; 11,435 matched nonsurgical patients (BMI ≥ 30)	All-cause mortality, coronary artery events, heart failure, nephropathy (10-year risk)	26 baseline variables (demographics, comorbidities, labs like HbA1c, meds)	Regression (Cox, exponential, Fine-Gray) and Random Forest; stratified by treatment	IDC outperformed RECODe for mortality, HF, nephropathy; no external validation performed	AUCs: Mortality 0.79/0.81; CAD 0.66/0.67; HF 0.73/0.75; Nephropathy 0.73/0.76 (surgical/nonsurgical)
Loïc Djaileb et al. [19]	2020	France	Prospective cohort	168 T2D patients at high/very high cardiovascular risk undergoing stress/rest SPECT	Major adverse cardiac events (MACE): cardiac death, MI, late revascularization	Stress MPE, SSS, ischemia extent, age, hypertension, stress ECG, rest MPE, others	Cox proportional hazards, Fine-Gray, LASSO, Random Forest; stress MPE added in nested models	Stress MPE added independent and incremental value beyond clinical & perfusion variables	AUC: 0.66–0.74 for MACEs; stress MPE HR = 2.77; C-statistic improved from 0.66 to 0.74
R. N. Guzder et al. [11]	2005	United Kingdom	Community-based prospective cohort	428 adults (aged 30–74) newly diagnosed with Type 2 Diabetes; no evident CVD at baseline	Cardiovascular disease (CVD), coronary heart disease (CHD)	Demographics, lipids, HbA1c, BP, ECG-LVH (for Framingham); diabetes duration for UKPDS	Risk prediction using Framingham risk function vs. UKPDS Risk Engine	Framingham worse at calibration; UKPDS included diabetes-specific variables	Framingham: CVD AUC = 0.673, CHD AUC = 0.657, underestimates events by ~33%; UKPDS: CHD AUC = 0.670, underestimates by 13%, better calibration
Junho Hyun et al. [21]	2023	South Korea	Retrospective cohort	589 asymptomatic adults with T2D, no CAD history, underwent CCTA	Cardiac death, nonfatal MI, unstable angina hospitalization, late revascularization	UKPDS risk category, CCTA stenosis severity, coronary artery calcium score (CACS)	Cox regression (unadjusted and adjusted), Kaplan–Meier, Harrell’s c-index, NRI, IDI	CCTA provided additive prognostic value over UKPDS; strongest in intermediate/high risk	UKPDS alone c = 0.658; adding CCTA stenosis: c = 0.724 (Δ = 0.066, *p* = .004); NRI = 0.190
Keishi Ichikawa et al. [18]	2021	Japan	Prospective cohort	529 T2DM outpatients with suspected coronary artery disease; no CVD history	Cardiovascular events (CV death, nonfatal MI, stroke, late revascularization, HF hospitalization)	NAFLD (liver: spleen attenuation < 1.0), CACS, Framingham Risk Score, visceral fat, labs	Cox regression (univariate and multivariate), Kaplan–Meier, ROC, NRI/IDI, global χ^2^	NAFLD, CACS, and FRS were independent predictors; NAFLD significantly improved risk classification	C-statistic: FRS + CACS = 0.71; FRS + CACS + NAFLD = 0.80 (*p* = 0.005); NRI = 0.551; HR for NAFLD = 5.43
Mi Jun Keng et al. [20]	2022	United Kingdom	Retrospective cohort	T2D patients, mostly white (98%), mean age 64, no history of CVD; n = 14,569	MI, other IHD, stroke, CV death, other death (7-year cumulative incidence)	Inputs to UKPDS-OM2: age, sex, duration of diabetes, BP, HbA1c, BMI, lipids, eGFR	UKPDS-OM2: stochastic individual simulation model using 10,000 Monte Carlo reps	UKPDS-OM2 poorly calibrated for CV outcomes in modern T2D cohort; relative risk ok	Overpredicted: CV death +269%, MI +149%, stroke +42%, other death +52%; c-statistic: MI = 0.58, IHD = 0.60, CV death = 0.70, stroke = 0.66
Yujian Li et al. [14]	2025	United Kingdom (UK Biobank)	Retrospective cohort	3335 T2D patients (1084 with incident CHD) from UK Biobank; controls n = 49,679	Coronary heart disease (CHD) in patients with T2D	Nine plasma proteins: PCSK9, NRP1, CD27, REN, RNASE1, ANGPTL4, PALM, RNASE6, CYTL1	Lasso regression; Cox model; Mendelian randomization; nomogram; ML integrated	Model 4 (demographics + 9 proteins) showed best performance and net clinical benefit	AUC = 0.819, C-index = 0.818 for full model (base + 9 proteins); base model alone AUC = 0.729
Luo et al. [22]	2024	Germany	Retrospective cohort	1492 discovery; 888 validation; stratified by T2D status	Incident coronary heart disease (non-fatal MI, coronary death, sudden death)	233 plasma proteins (Olink PEA) + Framingham risk factors; Priority-Lasso selected 4 (T2D) or 12 (non-T2D) proteins	Priority-Lasso Cox; 5-fold CV; basic Framingham block + protein block	Improves Framingham-based prediction	ΔC-index: +0.017 (T2D), +0.054 (non-T2D)
Lyu et al. [17]	2020	China	Retrospective cohort	456 T2DM patients; 270 without ACS, 186 with ACS	New-onset acute coronary syndrome (STEMI/NSTEMI/UA)	32 clinical & lab vars; final: age, BMI, DM duration, SBP, DBP, LDL-C, SUA, Lp(a), HTN hx, alcohol use	LASSO variable selection + multivariable logistic regression; nomogram; 70/30 split	Model showed good fit and net benefit	AUC: 0.830 training; 0.827 validation
Mordi et al. [13]	2024	United Kingdom	Retrospective cohort	10,556 individuals with T2D; no prior CV hospitalizations	Major adverse CV events (CV death, non-fatal MI, non-fatal stroke) at 10 y	Pooled Cohort Equation variables + genome-wide CHD polygenic risk score (~6.6 M SNPs)	Cox regression; compare PCE vs. PCE + PRS; internal evaluation (AUC, NRI, IDI)	Model improves MACE prediction over PCE alone	AUC = 0.722; NRI = 0.512; IDI = 0.034
Yeboah et al. [12]	2014	United States and Germany	Retrospective cohort	1343 adults with T2DM, no prior CVD (551 HNR, 792 MESA)	Incident hard CHD (fatal/non-fatal MI)	Age, sex, SBP, duration of diabetes, log(CAC + 25) ± ABI, hs-CRP, CIMT, FHx, smoking	Bayesian model averaging, Cox regression; internal bootstrap (100) + train/val split	Better than FRS/UKPDS; reclassification metrics support value	Model B AUC = 0.76; NRI = 0.19 vs. FRS, 0.22 vs. UKPDS
Zhang et al. [23]	2024	United Kingdom	Retrospective cohort	23,685 adults with T2DM (aged 40–71), 63.5% male, 87.3% White	External validation for all-cause mortality, CV-mortality, CHF, MI, stroke, IHD	Variables per 19 risk equations (RECODe, UKPDS-OM2, SCORE, Framingham, HKU-SG, etc.)	No new model; systematic external validation of 19 equations	RECODe best overall; UKPDS OM2 good for mortality; calibration varies	RECODe CHF: c = 0.71; UKPDS CV mortality: c = 0.71; Framingham CHF: c = 0.62

**Table 2:** PROBAST assessment.

Study	Design	Primary Outcome	Validation	Discrimination (AUC/C-Index)	RoB: Participants	RoB: Predictors	RoB: Outcome	RoB: Analysis	RoB: Overall	Applicability: Participants	Applicability: Predictors	Applicability: Outcome	Applicability: Overall	Key Notes
Abas 2025 [15] (Malaysia registry)	Development (ML)	Ischaemic heart disease (IHD) among T2D complications	Internal only	IHD ≈ 0.66 (others 0.71–0.84)	L	L	S	H	H	S	L	S	S	Large national cohort (~90k). Multiple complications; no external validation; registry-defined outcomes.
Aminian 2020 [16] (Cleveland Clinic)	Development (surgery vs. non-surgery; ML + Cox)	Coronary artery events (10-year), plus others	Internal (5-fold CV)	Coronary ≈ 0.66–0.67	S	L	S	H	H	H	L	S	H	Specialized surgical cohort; internal validation only; limited transportability.
Djaileb 2021 [19] (SPECT MPE)	Incremental (imaging added to clinical)	MACE (death/MI/revascularization)	Internal only	c-stat improved 0.66 → 0.74	S	L	L	H	H	H	S	S	S/H	Small high-risk cohort (n = 166, 44 events); risk of overfitting; requires SPECT.
Guzder 2005 [11] (UK; FRS & UKPDS)	External validation	CHD	External	0.657–0.670; poor calibration	L	L	L	S	S	L	L	L	L	Community, newly diagnosed T2D; classic scores underperform; calibration issues.
Hyun 2023 [21] (CCTA + UKPDS)	Incremental (imaging + risk score)	CAD composite (10-year)	Internal only	Δc ≈ +0.066 with stenosis; +0.039 with CACS	S	L	L	S	S	S	H	L	S/H	Asymptomatic T2D; requires CCTA; moderate gain over UKPDS.
Ichikawa 2021 [18] (NAFLD-CT + CACS)	Incremental (hepatic imaging + CACS)	CVD (incl. MI, revasc, stroke, HF)	Internal only	c-stat 0.71 → 0.80 with NAFLD	L	S	L	S	S	S	H	S	S	Suspected CAD; imaging-dependent; limited events.
Keng 2022 [20] (ASCEND; UKPDS-OM2)	External validation	MI, IHD, stroke, CV/other death	External	MI c ≈ 0.58; marked overprediction	L	L	L	L	S	L	L	L	L/S	Large pragmatic trial cohort; robust validation; recalibration needed.
Li 2025 [14] (UK Biobank; proteomics)	Development/Incremental (proteins)	Incident CHD in T2D	Internal only	AUC ≈ 0.82	S	S	L	H	H	S	H	L	S/H	Platform-dependent biomarkers; no external validation; optimism risk.
Luo 2024 [22] (KORA; proteomics)	Development + External validation	Incident CHD (with and without T2D)	Internal + External (population-based)	ΔC-index ≈ +0.017 in T2D	L	S	L	S	S	S	H	L	S	Moderate events in T2D subgroup; external validation present; small incremental gain.
Lyu 2020 [17] (NW China; ACS nomogram)	Development (hospital)	New-onset ACS	Internal (split-sample)	AUC ≈ 0.83	H	S	L	H	H	S	L	L	S	Hospital-based, selection bias likely; internal validation only.
Mordi 2024 [13] (Polygenic Risk Score)	Incremental (genetics + clinical)	Incident CHD	External (population-based)	Incremental gain vs. clinical baseline	L	L	L	S	S	S	L	L	S	PRS adds value; ancestry dependence may limit transportability.
Yeboah 2014 [12] (MESA/HNR; new score)	Development (two cohorts)	Incident CHD	Internal (cross-cohort style)	Noted improvement vs. baselines	L	L	L	S	S	S	L	L	S	Multi-ethnic development; lacks independent external validation in pure T2D.
Zhang 2024 [23] (23,685 T2D)	External validation (multiple models)	CVD and mortality endpoints	External	Model-dependent; systematic comparison	L	L	L	L	L	L	L	L	L	Large-scale external validation; strong transportability; supports recalibration needs.

## Data Availability

The original contributions presented in this study are included in the article/Supplementary Material. Further inquiries can be directed to the corresponding authors.

## Supplementary Materials

The following supporting information can be downloaded at: https://www.mdpi.com/article/10.3390/diagnostics16050765/s1, Table S1: Search strategy for each database; PRISMA 2020 Checklist.

## References

[1] Zhang H., Shi H. (2025). Construction of a Prediction Model for Coronary Heart Disease in Type 2 Diabetes Mellitus: A Cross-Sectional Study. Sci. Rep..

[2] American Diabetes Association Professional Practice Committee 10 (2025). Cardiovascular Disease and Risk Management: Standards of Care in Diabetes-2025. Diabetes Care.

[3] Einarson T.R., Acs A., Ludwig C., Panton U.H. (2018). Prevalence of Cardiovascular Disease in Type 2 Diabetes: A Systematic Literature Review of Scientific Evidence from Across the World in 2007–2017. Cardiovasc. Diabetol..

[4] Rathod M.B., Moukthika S., Karikunnel A.J., Harika K., Talla P., Jalakam M. (2024). A Cross-Sectional Evaluation of Cardiovascular Risk Assessment in Type 2 Diabetes Mellitus Patients Using the Framingham Risk Score. Cureus.

[5] Kavaric N., Klisic A., Ninic A. (2018). Cardiovascular Risk Estimated by UKPDS Risk Engine Algorithm in Diabetes. Open Med..

[6] Moons K.G.M., de Groot J.A.H., Bouwmeester W., Vergouwe Y., Mallett S., Altman D.G., Reitsma J.B., Collins G.S. (2014). Critical Appraisal and Data Extraction for Systematic Reviews of Prediction Modelling Studies: The CHARMS Checklist. PLoS Med..

[7] Fernandez-Felix B.M., López-Alcalde J., Roqué M., Muriel A., Zamora J. (2023). CHARMS and PROBAST at Your Fingertips: A Template for Data Extraction and Risk of Bias Assessment in Systematic Reviews of Predictive Models. BMC Med. Res. Methodol..

[8] Page M.J., McKenzie J.E., Bossuyt P.M., Boutron I., Hoffmann T.C., Mulrow C.D., Shamseer L., Tetzlaff J.M., Akl E.A., Brennan S.E. (2021). The PRISMA 2020 Statement: An Updated Guideline for Reporting Systematic Reviews. BMJ.

[9] Snell K.I.E., Levis B., Damen J.A.A., Dhiman P., Debray T.P.A., Hooft L., Reitsma J.B., Moons K.G.M., Collins G.S., Riley R.D. (2023). Transparent Reporting of Multivariable Prediction Models for Individual Prognosis or Diagnosis: Checklist for Systematic Reviews and Meta-Analyses (TRIPOD-SRMA). BMJ.

[10] Moons K.G.M., Wolff R.F., Riley R.D., Whiting P.F., Westwood M., Collins G.S., Reitsma J.B., Kleijnen J., Mallett S. (2019). PROBAST: A Tool to Assess Risk of Bias and Applicability of Prediction Model Studies: Explanation and Elaboration. Ann. Intern. Med..

[11] Guzder R.N., Gatling W., Mullee M.A., Mehta R.L., Byrne C.D. (2005). Prognostic Value of the Framingham Cardiovascular Risk Equation and the UKPDS Risk Engine for Coronary Heart Disease in Newly Diagnosed Type 2 Diabetes: Results from a United Kingdom Study. Diabet. Med..

[12] Yeboah J., Erbel R., Delaney J.C., Nance R., Guo M., Bertoni A.G., Budoff M., Moebus S., Jöckel K.H., Burke G.L. (2014). Development of a New Diabetes Risk Prediction Tool for Incident Coronary Heart Disease Events: The Multi-Ethnic Study of Atherosclerosis and the Heinz Nixdorf Recall Study. Atherosclerosis.

[13] Mordi I.R., Li I., George G., McCrimmon R.J., Palmer C.N., Pearson E.R., Lang C.C., Doney A.S. (2024). Incremental Prognostic Value of a Coronary Heart Disease Polygenic Risk Score in Type 2 Diabetes. Diabetes Care.

[14] Li Y., Li D., Lin J., Zhou L., Yang W., Yin X., Xu C., Cao Z., Wang Y. (2025). Proteomic Signatures of Type 2 Diabetes Predict the Incidence of Coronary Heart Disease. Cardiovasc. Diabetol..

[15] Abas M.Z., Li K., Choo W.Y., Wan K.S., Hairi N.N. (2025). Machine Learning Models for Predicting Type 2 Diabetes Complications in Malaysia. Asia Pac. J. Public Health.

[16] Aminian A., Zajichek A., Arterburn D.E., Wolski K.E., Brethauer S.A., Schauer P.R., Nissen S.E., Kattan M.W. (2020). Predicting 10-Year Risk of End-Organ Complications of Type 2 Diabetes with and Without Metabolic Surgery: A Machine Learning Approach. Diabetes Care.

[17] Lyu J., Li Z., Wei H., Liu D., Chi X., Gong D.W., Zhao Q. (2020). A Potent Risk Model for Predicting New-Onset Acute Coronary Syndrome in Patients with Type 2 Diabetes Mellitus in Northwest China. Acta Diabetol..

[18] Ichikawa K., Miyoshi T., Osawa K., Miki T., Toda H., Ejiri K., Yoshida M., Nanba Y., Yoshida M., Nakamura K. (2021). Prognostic Value of Non-Alcoholic Fatty Liver Disease for Predicting Cardiovascular Events in Patients with Diabetes Mellitus with Suspected Coronary Artery Disease: A Prospective Cohort Study. Cardiovasc. Diabetol..

[19] Djaileb L., Seiller A., Canu M., De Leiris N., Martin A., Poujol J., Fraguas-Rubio A., Leenhardt J., Carabelli A., Calizzano A. (2021). Prognostic Value of SPECT Myocardial Perfusion Entropy in High-Risk Type 2 Diabetic Patients. Eur. J. Nucl. Med. Mol. Imaging.

[20] Keng M.J., Leal J., Mafham M., Bowman L., Armitage J., Mihaylova B. (2022). Performance of the UK Prospective Diabetes Study Outcomes Model 2 in a Contemporary UK Type 2 Diabetes Trial Cohort. Value Health.

[21] Hyun J., Lee P.H., Lee J., Yang Y., Kim J.H., oh Kim T., Kang S.-J., Kim J.K., Lee J.S., Lee S.-W. (2023). Ten-Year Prognostic Value of Coronary CT Angiography in Asymptomatic Patients with Type 2 Diabetes. Rev. Española Cardiol. (Engl. Ed.).

[22] Luo H., Huemer M.T., Petrera A., Hauck S.M., Rathmann W., Herder C., Koenig W., Hoyer A., Peters A., Thorand B. (2024). Association of Plasma Proteomics with Incident Coronary Heart Disease in Individuals with and Without Type 2 Diabetes: Results from the Population-Based KORA Study. Cardiovasc. Diabetol..

[23] Zhang Y., Jiong O.X., Tang S., Tang Y.C., Wong C.T., Ng C.S., Quan J. (2024). Comparison of Prediction Models for Cardiovascular and Mortality Risk in People with Type 2 Diabetes: An External Validation in 23 685 Adults Included in the UK Biobank. Diabetes Obes. Metab..

[24] Wang M., Zhang W., Li J., Luan Y., Ding X., Hu Y. (2025). Development and Validation of a Nomogram Prediction Model for Coronary Heart Disease in Diabetic Patients: A Study Based on the 2011–2020 NHANES Database. BMC Cardiovasc. Disord..

[25] Polonsky T.S., McClelland R.L., Jorgensen N.W., Bild D.E., Burke G.L., Guerci A.D., Greenland P. (2010). Coronary Artery Calcium Score and Risk Classification for Coronary Heart Disease Prediction. JAMA.

[26] Ganna A., Salihovic S., Sundström J., Broeckling C.D., Hedman Å.K., Magnusson P.K.E., Pedersen N.L., Larsson A., Siegbahn A., Zilmer M. (2014). Large-Scale Metabolomic Profiling Identifies Novel Biomarkers for Incident Coronary Heart Disease. PLoS Genet..

[27] Basu S., Sussman J.B., Berkowitz S.A., Hayward R.A., Bertoni A.G., Correa A., Mwasongwe S., Yudkin J.S. (2018). Validation of Risk Equations for Complications of Type 2 Diabetes (RECODe) Using Individual Participant Data from Diverse Longitudinal Cohorts in the U.S. Diabetes Care.

[28] Pagano E., Konings S.R.A., Di Cuonzo D., Rosato R., Bruno G., van der Heijden A.A., Beulens J., Slieker R., Leal J., Feenstra T.L. (2021). Prediction of Mortality and Major Cardiovascular Complications in Type 2 Diabetes: External Validation of UK Prospective Diabetes Study Outcomes Model Version 2 in Two European Observational Cohorts. Diabetes Obes. Metab..

[29] Valipour M., Khalili D., Solaymani-Dodaran M., Motevalian S.A., Khamseh M.E., Baradaran H.R. (2023). External Validation of the UK Prospective Diabetes Study (UKPDS) Risk Engine in Patients with Type 2 Diabetes Identified in the National Diabetes Program in Iran. J. Diabetes Metab. Disord..

[30] Zhang X.L., Wan G., Yuan M.X., Yang G.R., Fu H.J., Zhu L.X., Xie R.R., Lv Y.J., Zhang J.D., Li Y.L. (2020). Improved Framingham Risk Scores of Patients with Type 2 Diabetes Mellitus in the Beijing Community: A 10-Year Prospective Study of the Effects of Multifactorial Interventions on Cardiovascular Risk Factors (The Beijing Communities Diabetes Study 22). Diabetes Ther..

[31] León-Figueroa D.A., Barboza J.J., Valladares-Garrido M.J., Sah R., Rodriguez-Morales A.J. (2024). Prevalence of intentions to receive monkeypox vaccine. A systematic review and meta-analysis. BMC Public Health.

[32] León-Figueroa D.A., Barboza J.J., Siddiq A., Sah R., Valladares-Garrido M.J., Rodriguez-Morales A.J. (2024). Knowledge and attitude towards mpox: Systematic review and meta-analysis. PLoS ONE.

[33] Swarup S.S., P. A.K., Padhi B.K., Satapathy P., Shabil M., Bushi G., Gandhi A.P., Khatib M.N., Gaidhane S., Zahiruddin Q.S. (2024). Cardiovascular consequences of financial stress: A systematic review and meta-analysis. Curr. Probl. Cardiol..

[34] Sandeep M., Padhi B.K., Yella S.S.T., Sruthi K.G., Venkatesan R.G., Sasanka K.S.B.S.K., Satapathy P., Mohanty A., Al-Tawfiq J.A., Iqhrammullah M. (2023). Myocarditis manifestations in dengue cases: A systematic review and meta-analysis. J. Infect. Public Health.

